# Infektiologie in der stationären Versorgung in Deutschland: Positionspapier der Deutschen Gesellschaft für Infektiologie (DGI)

**DOI:** 10.3205/000319

**Published:** 2023-05-26

**Authors:** Gerd Fätkenheuer, Leif Erik Sander, Hortense Slevogt, Bernd Salzberger, Deutsche Gesellschaft für Infektiologie

**Affiliations:** 1 Infektiologie, Medizinische Klinik I, Universitätsklinikum Köln, Deutschland; 2 Klinik für Infektiologie und Intensivmedizin, Charité – Universitätsmedizin Berlin, Deutschland; 3 Klinische Infektiologie, Klinik für Pneumologie, MH Hannover, Deutschland; 4 Krankenhaushygiene und Infektiologie, Universitätsklinikum Regensburg, Deutschland; 5 Berlin, Deutschland

**Keywords:** infectious disease medicine, bacteremia, immunocompromised host, Infektiologie, Bakteriämie, immunkompromittierte Patienten

## Abstract

Spezifische infektiologische Kompetenz verbessert die stationäre Versorgung von Patienten mit Infektionskrankrankheiten. Mit der neuen Facharztbezeichnung Innere Medizin und Infektiologie wird diese Expertise auch in Deutschland zugänglich. Die strukturelle Einbindung der Infektiologie und die Definition einer Leistungsgruppe in Kliniken der Level 2 und 3 werden dargestellt.

## Abstract (en)

Clinical expertise in infectious diseases is crucial in treating patients with infectious complications. The new board certification in infectious diseases will establish this expertise in Germany. The role of the specialty of infectious diseases in German hospitals and the definition for clinical services in hospitals (levels 2 and 3) are outlined here.

## Infektionskrankheiten – häufig und prognostisch relevant

Infektionskrankheiten sind häufige Diagnosen in deutschen Krankenhäusern. In den Jahren 2021/2022 betrug der Anteil von Infektionskrankheiten an Hauptdiagnosen 12 bis 13%; dies entspricht etwa 2 Millionen stationären Behandlungen (eigene Berechnungen, basierend auf InEK-Daten [1] (Tabelle 1 (Tab. 1)). Bei zusätzlicher Berücksichtigung von Nebendiagnosen machen Infektionen mehr als 20% aller Behandlungsfälle aus [2]. Die Komplexität von Infektionserkrankungen stellt hohe Anforderungen an die Behandlung [3]. So ist der Anteil von Infektionen an den Hauptdiagnosen auf Intensivstationen in den letzten Jahrzehnten stetig angewachsen [4]. Behandlungen in der Onkologie, der Chirurgie oder der Transplantationsmedizin werden immer komplexer und steigern damit auch das Risiko für infektiologische Komplikationen. Parallel dazu steigt der Anteil weiterer vulnerabler Patientengruppen wie immunsupprimierten oder auch hochbetagten Menschen. 

Für die optimale Behandlung dieser Patienten wird eine spezifische infektiologische Expertise benötigt. Zahlreiche Studien haben gezeigt, dass die Einbeziehung von Infektiologen zum Beispiel bei Blutstrominfektionen, Pilzinfektionen oder Sepsis die Mortalität, Krankenhausverweildauer und Behandlungskosten senkt [5]. Infektiologische Expertise ist bei der Behandlung von Patienten mit Infektionen durch multiresistente Erreger (MRE) zwingend notwendig. Sie trägt dazu bei, die Verbreitung von MRE zu verhindern bzw. einzudämmen, und sie wird in der Bewältigung von Epidemien und Pandemien dringend benötigt.

## Aktuelle Situation der Infektiologie in Deutschland

Mit der Einführung der Facharztbezeichnung für Innere Medizin und Infektiologie 2021 wurde eine wesentliche strukturelle Voraussetzung für eine Verbesserung der Versorgung von Menschen mit Infektionskrankheiten geschaffen. Bis dahin gab es lediglich eine Zusatzweiterbildung (ZWB) Infektiologie für Fachärzte mehrerer Disziplinen. Als Ausdruck des großen Bedarfs in diesem Bereich nahm die Zahl der Ärzte mit dieser Zusatzweiterbildung rasch zu (2014: 647 Weiterbildungen; 2021: 948 Weiterbildungen) [6]. Zugleich stieg die Zahl der Ärzte, die eine spezielle Fortbildung in der rationalen Anwendung von antimikrobiellen Substanzen (*Antibiotic Stewardship*, ABS) durchlaufen haben (bis 2022: >1.000 Fortbildungen) [7], und in vielen Kliniken wurden ABS-Teams etabliert.

Auch wenn noch lange nicht alle größeren Krankenhäuser eine Abteilung mit infektiologischem Schwerpunkt aufweisen, ist die Zahl solcher Kliniken stetig gewachsen. Meistens sind die infektiologischen Bereiche Teil von internistischen Abteilungen mit weiteren Schwerpunkten (z.B. Gastroenterologie, Hämatologie/Onkologie, Pneumologie). Fachabteilungen mit besonders hoher Expertise in der Infektiologie können sich von der Deutschen Gesellschaft für Infektiologie als „Zentrum für Infektiologie (DGI)“ zertifizieren lassen. Die derzeit 32 zertifizierten Kliniken bzw. Abteilungen können als Multiplikatoren für die rasche Weiterbildung von Fachärzten für Innere Medizin und Infektiologie angesehen werden. 

Ein aktuelles Problem besteht in der Abbildung infektiologischer Leistungen im *Diagnosis-Related-Groups*(DRG)-System. Infektionen sind typischerweise Bestandteil des Krankheitsspektrums aller Organsysteme und werden in aller Regel in den organspezifischen Leistungsgruppen, in der Allgemeinen Inneren Medizin oder der Chirurgie abgebildet. Für die Infektiologie sind nur wenige Operationen- und Prozedurenschlüssel (OPS-Codes) definiert, da es keine spezifischen oder exklusiven apparativen Leistungen des Fachgebiets gibt. 

## Bessere Behandlung von Infektionspatienten durch Ausbau der Strukturen

Die Verbesserung der infektiologischen Versorgung soll im Rahmen der bevorstehenden Krankenhausreform auf mehreren Ebenen erfolgen. Die höchste fachliche Kompetenz liegt bei Fachärzten für Innere Medizin und Infektiologie, gefolgt von Fachärzten mit Zusatzweiterbildung Infektiologie und von Fachärzten mit ABS-Fortbildung. Bettenführende infektiologische Abteilungen sollten in allen Kliniken der Maximalversorgung (entsprechend Level 3 nach Umsetzung der Krankenhausreform) vorgehalten werden (optional auch bei Level 2). Für die Behandlung kontagiöser Patienten müssen geeignete Isolierzimmer vorhanden sein und es muss ausreichend geschultes Personal zur Verfügung stehen. 

Auf ärztlicher Seite sind hier drei Fachärzte für Innere Medizin und Infektiologie in Vollzeit anzusetzen (Tabelle 2 (Tab. 2)). Da diese Facharztbezeichnung erst im Jahr 2021 eingeführt wurde und seit Ende 2022 in den Landesärztekammern sukzessive umgesetzt wird, ist eine Übergangszeit von acht Jahren notwendig. In diesem Zeitraum wird es möglich sein, die erforderliche Zahl von Fachärzten weiterzubilden. In der Übergangszeit können Fachärzte für Innere Medizin mit einjähriger Zusatzweiterbildung Infektiologie die vorhandene Lücke schließen. Langfristig ist jedoch der Einsatz von Fachärzten für Innere Medizin und Infektiologie notwendig, da die Anforderungen an die Behandlung von Patienten mit komplexen Infektionskrankheiten nur in einer längeren Weiterbildungszeit sicher erworben werden können. Ein Zertifizierungsprozess für infektiologische Abteilungen ist bereits durch die DGI etabliert (siehe oben). Diese Zertifizierung kann an die neuen Voraussetzungen angepasst und sollte dann von allen infektiologischen Abteilungen des Level 3 (fakultativ auch Level 2) erbracht werden.

Zudem ist bisher keine spezifische Pflegeweiterbildung für die Betreuung von Patienten mit Infektionskrankheiten vorhanden. Diese Expertise soll in den kommenden Jahren definiert und etabliert werden. 

## Definition einer Leistungsgruppe „Infektiologie“

Die Leistungsgruppe „Infektiologie“ beinhaltet besonders komplizierte und schwere Infektionen bzw. Infektionen bei Patienten mit besonderen Risikofaktoren, für die spezielle personelle und strukturelle Voraussetzungen vorhanden sein müssen. Das Krankheitsspektrum kann aufgrund der Vielzahl der Infektionserreger (ICD A00-B99) am besten anhand der spezifischen Inhalte des jeweiligen Gebiets nach der Weiterbildungsordnung (WBO) abgegrenzt werden. Es ist unmittelbar evident, dass bei der hohen Zahl von stationär behandelten Infektionen eine eigene Leistungsgruppe ein hohes Volumen an Diagnosen haben wird. Dies gilt auch dann, wenn man eine strenge Definition für Fälle mit einer hohen Komplexität zugrunde legt (z.B. 5% oder 2,5% der Fälle).

Die Etablierung der Leistungsgruppe „Infektiologie“ soll Voraussetzung sein für Leistungsgruppen anderer Fachbereiche, in denen gehäuft komplizierte Infektionserkrankungen vorkommen (Tabelle 3 (Tab. 3)). Zusätzliche OPS-Codes für infektiologische Beratung und für eine allgemeine infektiologische Komplexbehandlung sind beantragt bzw. in Bearbeitung.

ABS-Teams, entsprechend der Leitlinie „Strategien zur Sicherung rationaler Antibiotika-Anwendung“ beschrieben, sind in Kliniken jeden Levels erforderlich. In Kliniken ab 500 Betten ist hierfür eine ärztliche Vollzeitstelle notwendig. In den Krankenhäusern mit einer Leistungsgruppe „Infektiologie“ sollte das ABS-Team von einem Facharzt für Innere Medizin und Infektiologie geleitet werden (Level 3 obligatorisch, Level 2 fakultativ) [7].

Mit diesen Maßnahmen können Patienten mit Infektionskrankheiten optimal behandelt werden und die Kliniken werden gegenüber bereits bestehenden und neuen Herausforderungen im Bereich der Infektionsmedizin deutlich besser gerüstet sein.

## Anmerkungen

### Interessenkonflikte

Die Autoren erklären, dass sie keine Interessenkonflikte in Zusammenhang mit diesem Artikel haben.

## Figures and Tables

**Table 1:**
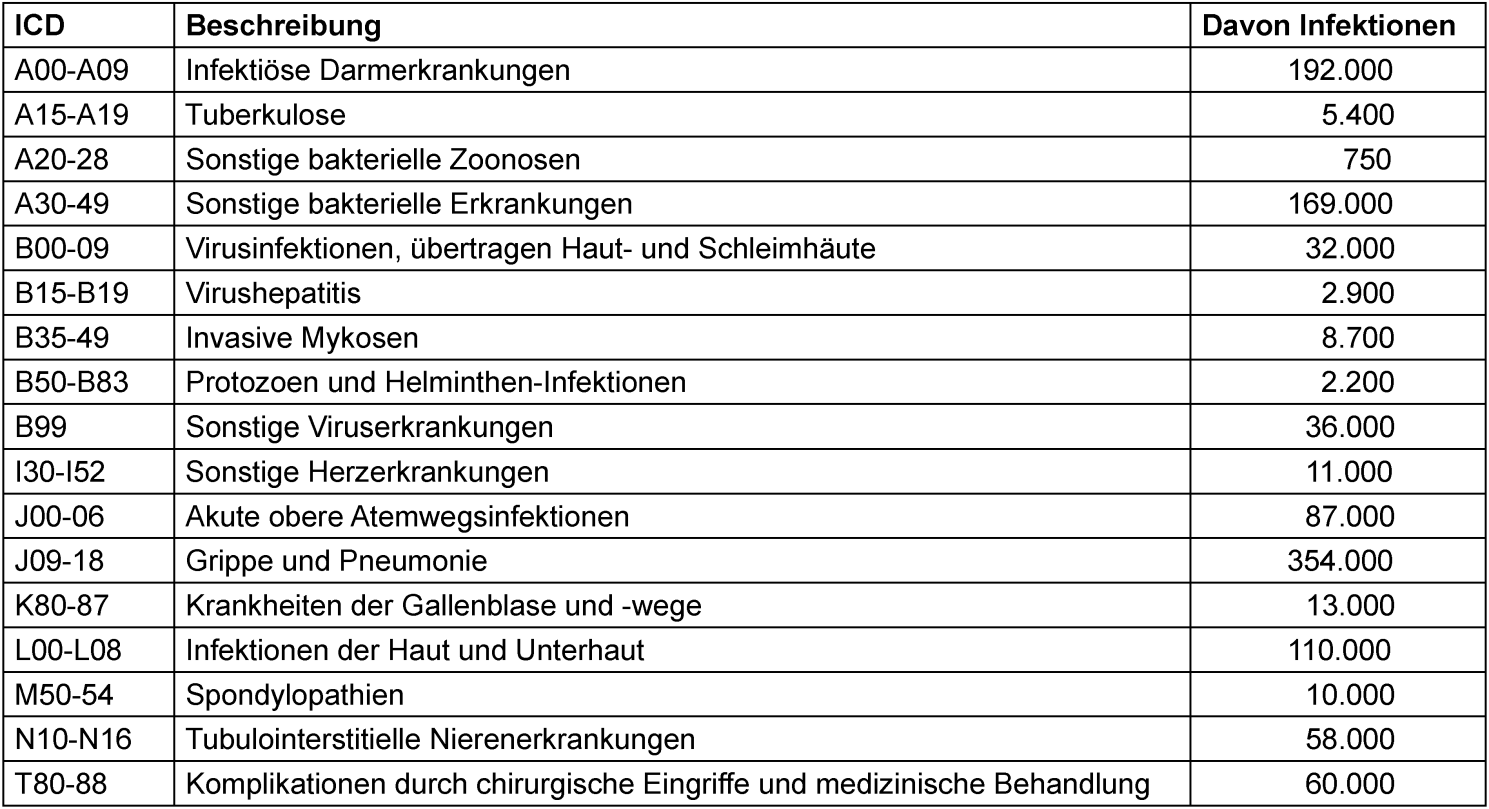
Tabelle 1: Stationäre Behandlungszahlen für Infektionskrankheiten nach ICD-Gruppen, Jahr 2021 (1,5 Millionen ausgewählte Behandlungen aus 2 Millionen), Daten nach InEK [1]

**Table 2:**
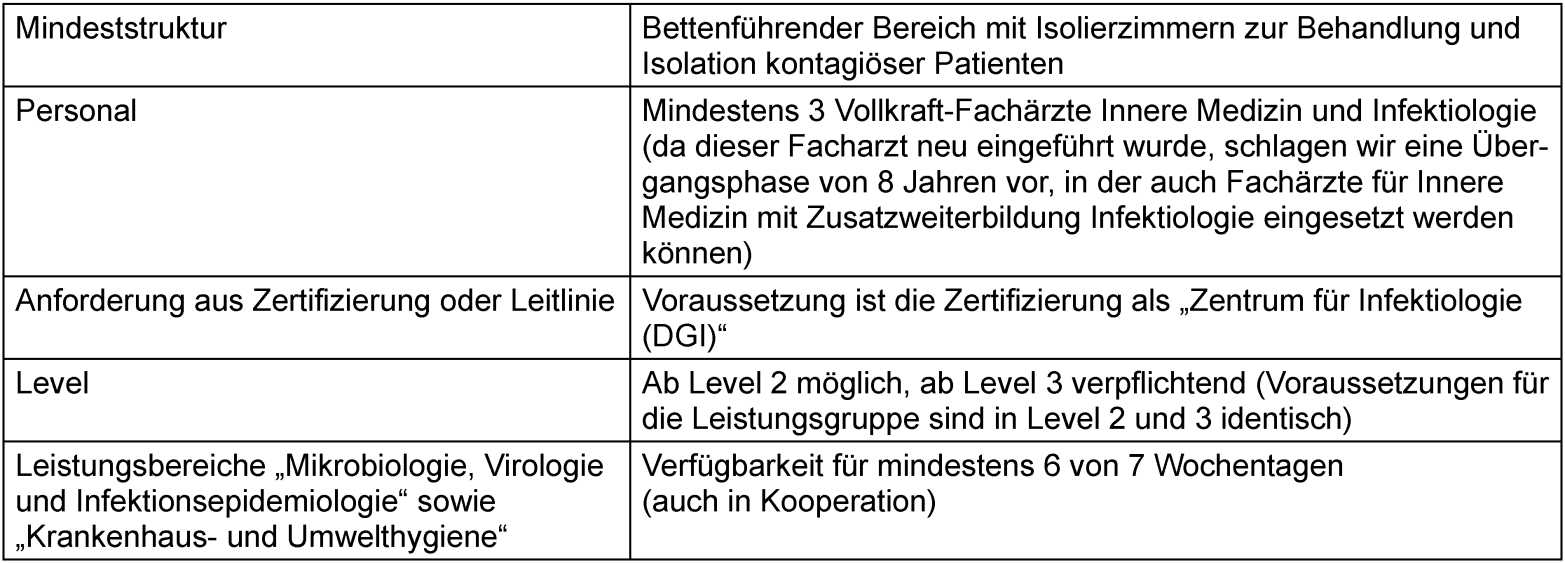
Tabelle 2: Strukturvoraussetzungen der Leistungsgruppe „Komplexe Infektiologie“ (identisch für Level 2 und 3)

**Table 3:**
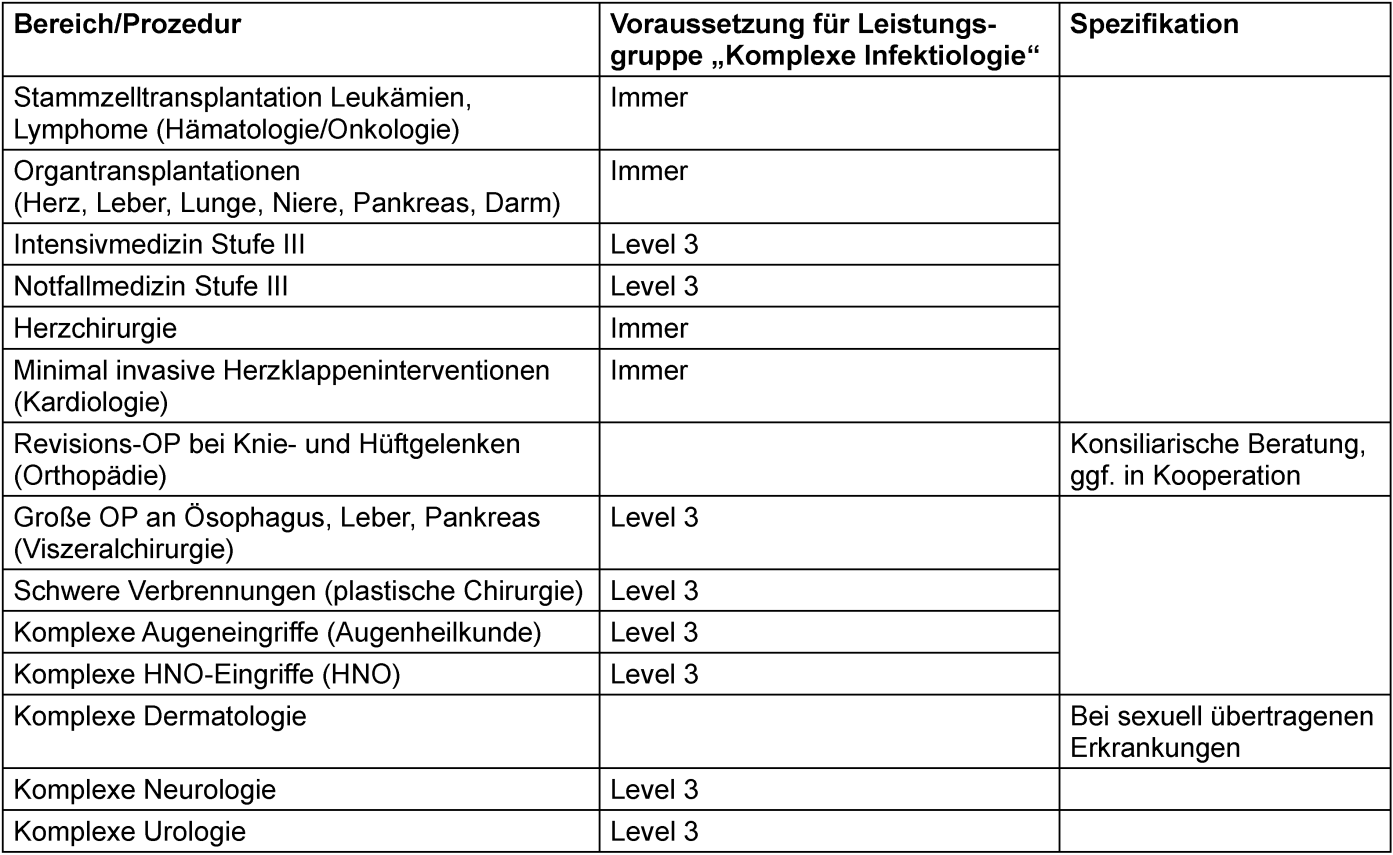
Tabelle 3: Vernetzung der Leistungsgruppe „Komplexe Infektiologie“ mit anderen Bereichen
